# Comprehensive Dental Management of a Rolandic Epilepsy Patient Under Local Anesthesia: A Case Report

**DOI:** 10.7759/cureus.37060

**Published:** 2023-04-03

**Authors:** Bader Fatani, Rania Kalantan, Alhanouf K Binhezaim, Sarah AlBluwi

**Affiliations:** 1 Dentistry, College of Dentistry, King Saud University, Riyadh, SAU; 2 Pediatric Dentistry, Department of Pedodontics Dental Center, Prince Sultan Military Medical City, Riyadh, SAU

**Keywords:** oral health, sedation, local anesthesia, dental management, rolandic epilepsy

## Abstract

Dental treatment of epilepsy patients is often challenging and requires careful consideration of their sudden movements. Epilepsy patients often require sedation (e.g., nitrous oxide or intravenous sedation) to receive their required dental treatments. Rolandic epilepsy (RE) is a specific type of epilepsy in children with certain electroencephalogram (EEG) abnormalities and motor focal seizures with no signs of neurological deficits. This report discusses a case of an RE patient who was treated comprehensively under local anesthesia with careful evaluation of the patient’s medical conditions.

## Introduction

Rolandic epilepsy (RE) is a specific type of epilepsy in children that is characterized by specific electroencephalogram (EEG) abnormalities and motor focal seizures with no signs of neurological deficits [1]. The seizures in RE develop from an area in the brain called the rolandic area [2]. The incidence of this epilepsy is 10-20 per 100,000 children and it persists up to the age of 15 years. RE accounts for approximately 15% of all epilepsy cases in children, making it the most common childhood epilepsy syndrome [3]. Its symptoms are usually unilateral and include facial stiffness and twitching, tingling, and numbness of the throat and face (i.e., lips, tooth, gums, tongue, and the inner side of the cheek), thereby leading to difficulty in speaking properly due to hypersalivation, gurgling noises, drooling, and speech arrest [3]. However, these symptoms may generally improve as the child reaches the age of 15 years. EEG will then start to normalize, and no developmental issues are generally observed [4]. This report presents a case of RE that was comprehensively treated under local anesthesia.

## Case presentation

**Figure 1:**
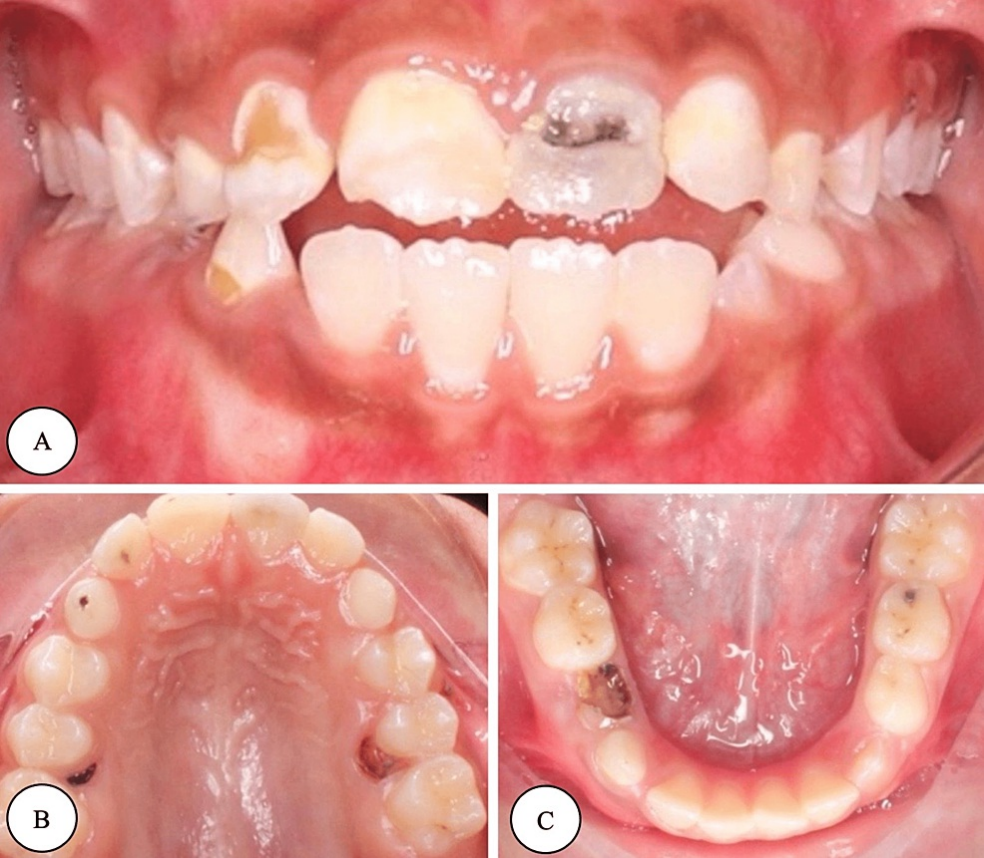
Preoperative photographs (A) Frontal view. (B) Occlusal maxillary view. (C) Occlusal mandibular view

**Figure 2:**
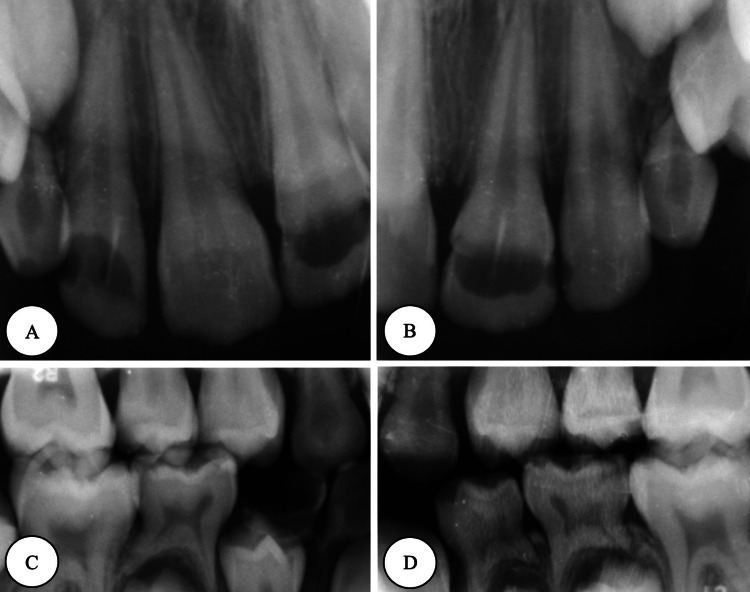
Preoperative radiographs (A) Periapical right-shifted radiograph. (B) Periapical left-shifted radiograph. (C) Right-side bitewing. (D) Left-side bitewing

The patient was a nine-year-old Saudi girl with a known case of RE and left polymicrogyria, with no known allergies, treated with carbamazepine (9 mL) twice per day. Her last seizure attack had been six months ago, occurring upon the cessation of carbamazepine. The patient presented to the dental clinic with a chief complaint of “multiple cavities in the upper front teeth.” Upon clinical examination, she was in the late mixed dentition stage with multiple carious teeth, poor oral hygiene, tongue thrusting habit with anterior open bite, and uncomplicated crown (enamel only) fracture in tooth #11. Her behavior regarding dental treatment was positive (Frankl rating: III) [5], and she was considered at high risk according to the Caries Risk Assessment Tool. Preoperative photographs and radiographs are presented in Figure 1 and Figure 2.

**Figure 3:**
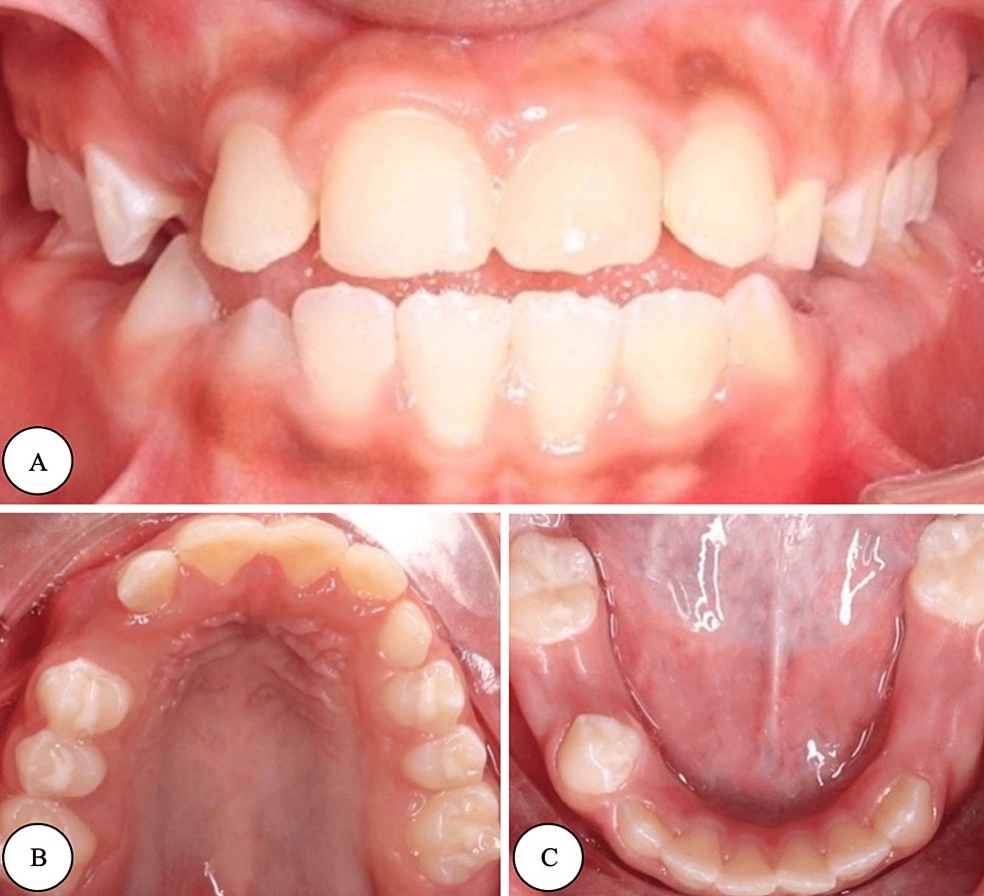
Postoperative photographs (A) Frontal view. (B) Occlusal maxillary view. (C) Occlusal mandibular view

**Figure 4:**
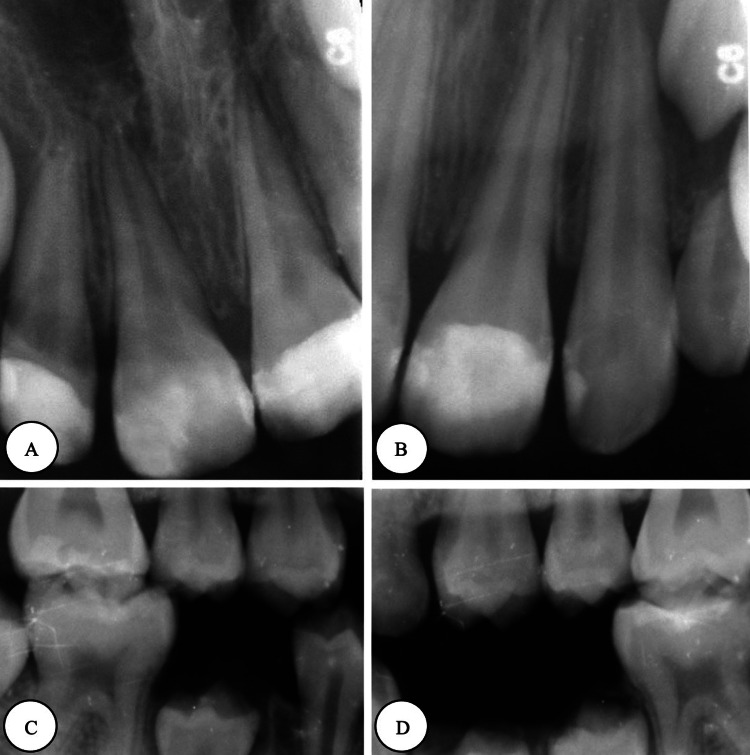
Postoperative radiographs (A) Periapical right-shifted radiograph. (B) Periapical left-shifted radiograph. (C) Right-side bitewing. (D) Left-side bitewing

**Figure 5:**
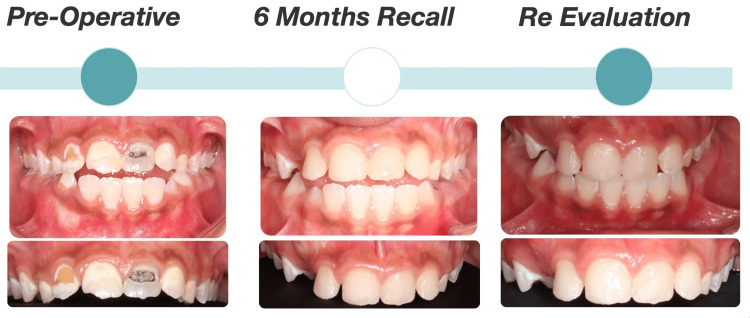
Case summary showing preoperative evaluation, six-month recall, and re-evaluation

After obtaining neurological clearance, full-mouth dental rehabilitation under local anesthesia (lidocaine) was performed, which included indirect pulp treatment for tooth #12, direct pulp capping for tooth #21, and aesthetic composite restoration for the upper anterior teeth. Simple restorative treatments were done for the posterior teeth. In addition, a palatal crib appliance was placed after orthodontic consultation to correct the anterior open bite, and a follow-up at one month revealed improvement in the anterior relationship. Postoperative photographs and radiographs are presented in Figure 3 and Figure 4. Case summaries showing preoperative evaluation, six-month recall, and re-evaluation are demonstrated in Figure 5.

## Discussion

In individuals with special healthcare needs, dental health is often neglected, which can lead to a deterioration in oral health. Patients with epilepsy and other similar conditions face additional challenges with their oral healthcare, as they tend to have higher occurrences of missing and decayed teeth, fewer restored or replaced teeth, and may experience side effects from anti-seizure medications. Most patients with well-controlled seizures can receive outpatient care, but it is crucial that dentists are educated on epilepsy and its related medications. Although a comprehensive medical history is essential for all dental patients, it is especially important for those with epilepsy, as they often have other medical and psychiatric disorders. Patients should ideally consult their neurologists to ensure tailored care. Neurologists and dentists should collaborate on finding the best approach by discussing seizure types, their frequency and severity, and the patient’s circadian periodicity to schedule dental visits during times when it is less likely for the patient to experience seizures [6]. A study by Subki et al. showed that most children with epilepsy reported having poor dental health and expressed a strong need for dental care [7]. A study by Mielnik-Błaszczak et al. assessed access to dental treatment for epilepsy patients [8], revealing that while most of the participants saw a general practitioner regularly, few children received standard dental checkups. Children who resided in larger cities visited the dentist more frequently than those in small towns or rural areas. Additionally, 46.73% of those surveyed faced obstacles in obtaining dental care for their children.

Good oral hygiene is essential in children with epilepsy. A study by Goyal et al. showed that consistent use of liquid medicines containing sucrose increases the chance of dental cavities in children with epilepsy [9]. Therefore, epileptic children who use these liquids for extensive periods should receive specialized and thorough preventative dental care if there are no other alternatives to these sugar-based medications. According to Yeung et al., children who suffer from epilepsy tend to have worse oral health, especially gum health, compared to those who do not have epilepsy [10]. Moreover, parents have a responsibility to monitor their children’s oral hygiene and maintain regular checkups with their dentist. Special care should be provided to epileptic patients to improve their oral health and reduce further dental problems [11].

RE can affect certain body parts like the face, mouth, and arms, and may cause speech issues [1]. Although RE usually does not directly cause dental issues, they may arise as a result of problems with speech disorders. Therefore, careful management of the patient's oral health and sudden movements while providing dental treatment is necessary. In general, epilepsy patients who take medication for their condition may experience negative effects on their oral health, which can make dental procedures or their underlying conditions more complicated. These medications can lead to conditions such as gingival hyperplasia, stomatitis, or xerostomia [6]. In case conscious sedation or general anesthesia is required due to difficult child movements, identifying the severity, frequency, type, and triggering factors of epilepsy is crucial. Anesthesia can create the best conditions for dental treatment for patients with special needs. It is also important to take into account any psychological, physical, and social concerns that the patient may have [12,13].

## Conclusions

Managing patients with medical conditions combined with dental aesthetic problems is challenging yet rewarding. Epilepsy patients often require sedation for dental treatment. In this report, we discussed a case of a child with RE who was fully treated under local anesthesia. Aesthetic restoration of upper anterior teeth can be especially challenging due to the child’s behavior. The advent of various advanced techniques, devices, and materials has helped children and adolescents improve their self-image through better dental restoration. Moreover, the clinician should have enough knowledge regarding the proper management of patients with special needs as well as the associated complications to ensure favorable treatment outcomes.
